# Bilateral Deep Vein Thrombosis in a Young Man With Inferior Vena Cava Agenesis: A Case Report

**DOI:** 10.7759/cureus.107649

**Published:** 2026-04-24

**Authors:** Hadeel AlBayyat, Yacoub Abuzied, Anfal AlShaya, Mohammed AlShaikh, Mohammed AlSheef

**Affiliations:** 1 Internal Medicine, King Fahad Medical City, Riyadh, SAU; 2 Nursing, King Fahad Medical City, Riyadh, SAU; 3 Interventional Radiology, King Fahad Medical City, Riyadh, SAU; 4 Internal Medicine and Thrombosis, King Fahad Medical City, Riyadh, SAU

**Keywords:** back pain, bilateral dvt, ivc agenesis, pe, unprovoked dvt

## Abstract

Inferior vena cava (IVC) agenesis is a rare congenital vascular anomaly that may predispose young individuals to venous thromboembolism in the absence of classical risk factors. We report the case of a 35-year-old man, an active smoker with no significant medical history, who presented with a 10-day history of bilateral lower limb swelling and severe back pain radiating to the lower limbs.

Doppler ultrasonography demonstrated extensive bilateral deep vein thrombosis (DVT), involving the right external iliac, common femoral, and femoral veins, with complete thrombosis of the left lower limb venous system extending to the posterior tibial veins. The patient was initiated on therapeutic anticoagulation with enoxaparin (1 mg/kg twice daily). Contrast-enhanced computed tomography (CT) of the abdomen failed to visualize the subhepatic IVC and revealed thrombosis in the left hemiazygos vein and bilateral external iliac veins, raising suspicion for IVC agenesis. CT pulmonary angiography confirmed bilateral lower segmental pulmonary emboli.

Echocardiography showed no evidence of right ventricular strain but revealed an incidental bicuspid aortic valve (type 1 Sievers) with mild aortic stenosis and regurgitation. The patient was subsequently transitioned from enoxaparin to fondaparinux and later to a direct oral anticoagulant for long-term management.

This case highlights the importance of considering congenital venous anomalies such as IVC agenesis in young patients presenting with extensive or bilateral DVT, particularly in the absence of traditional risk factors. Early recognition is essential for guiding appropriate long-term anticoagulation and reducing the risk of recurrent thromboembolic events.

## Introduction

Inferior vena cava (IVC) agenesis is a rare congenital vascular anomaly resulting from abnormal embryological development and is characterized by partial or complete absence of the IVC. It is estimated to occur in less than 1% of the general population [1]. In most cases, the condition remains clinically silent and is often identified incidentally during imaging performed for unrelated indications. However, in the absence of a normal IVC, venous return is redirected through collateral pathways, leading to venous stasis and an increased risk of thrombotic events, particularly deep vein thrombosis (DVT), even in otherwise healthy young individuals [2].

Although IVC agenesis is frequently an isolated finding, it has been associated with other congenital anomalies, including cardiac, renal, splenic, and pulmonary abnormalities [3]. Early recognition is important, as it has implications for long-term management, particularly in preventing recurrent thrombotic events. Management is primarily conservative and focuses on anticoagulation and symptom control, as surgical options remain limited. Diagnosis is typically confirmed using contrast-enhanced computed tomography (CT) or magnetic resonance imaging, which can identify the absence of the IVC and collateral venous pathways [4]. Delayed diagnosis may lead to complications such as recurrent thrombosis and chronic venous insufficiency [5].

Low back or flank pain is an uncommon presentation of bilateral external iliac vein thrombosis. However, when such symptoms are accompanied by lower limb swelling, DVT should be considered [6]. Some cases have been reported to mimic sciatica, with radiating leg pain, or even resemble lumbar disc herniation [7]. These overlapping features can delay diagnosis and appropriate management. Accordingly, clinicians should maintain a high index of suspicion and consider vascular etiologies when evaluating atypical lower back pain with associated limb symptoms.

We report a rare case of IVC agenesis presenting with bilateral lower limb thrombosis and atypical back pain, highlighting the diagnostic challenges and the importance of early recognition in preventing complications.

This work was previously presented as a meeting abstract at the ISTH 2025 Congress Annual Scientific Meeting, held in Washington, DC, USA, from June 21 to June 25, 2025 (abstract no. PB0450).

## Case presentation

This case report describes a 35-year-old man, an active cigarette smoker with no known prior medical conditions, who presented to the Emergency Department of a tertiary care center with a 10-day history of bilateral lower limb swelling, associated with significant right-sided flank and back pain. Approximately two weeks prior to symptom onset, the patient had undertaken a continuous five-hour car journey. He denied any history of chest pain, dyspnea, syncope, palpitations, fever, joint pain, skin rash, mucosal ulcers, or constitutional symptoms.

There was no history of recent trauma, surgery, hospitalization, prolonged immobilization, or exposure to new medications, herbal supplements, or recent vaccinations. The patient worked in an office-based role with a predominantly sedentary lifestyle. Family history was notable for a provoked venous thromboembolism (VTE) event in his sister during long-distance travel in her fourth decade of life. There was no known family history of malignancy, autoimmune disease, or inherited thrombophilia.

On examination, the patient was alert and hemodynamically stable. His blood pressure was 130/76 mmHg, heart rate 100 beats/minute, and oxygen saturation 99% on room air. Cardiopulmonary examination was unremarkable. However, the lower limb examination revealed bilateral nontender pitting edema extending to the mid-thigh. Peripheral pulses were intact, and capillary refill time was less than two seconds, with no evidence of acute circulatory compromise.

**Figure 1:**
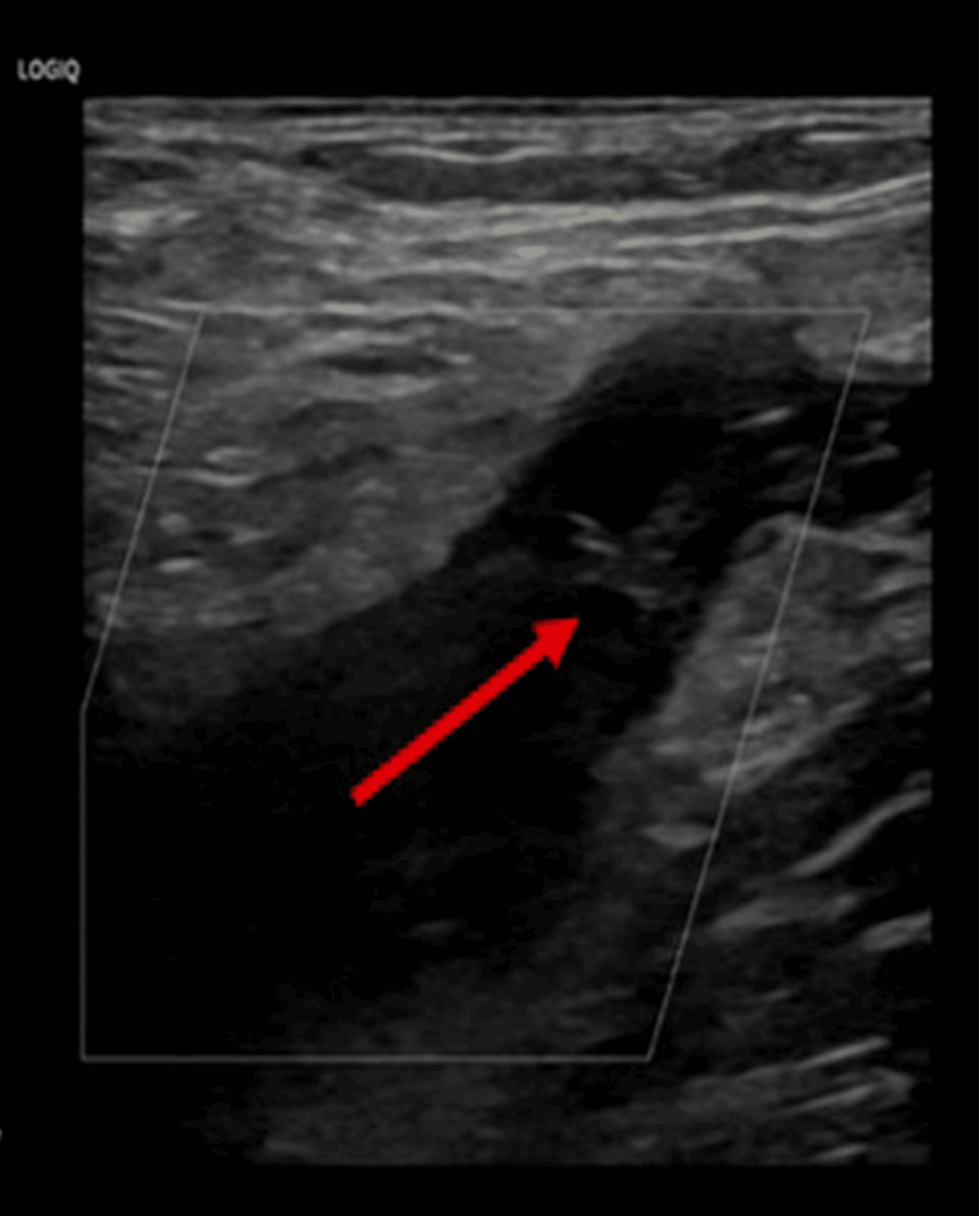
Doppler ultrasound of the right CFV showing intraluminal echogenic thrombus (arrow) with loss of compressibility, consistent with acute deep vein thrombosis involving the right external iliac, common femoral, and femoral veins CFV: common femoral vein

**Figure 2:**
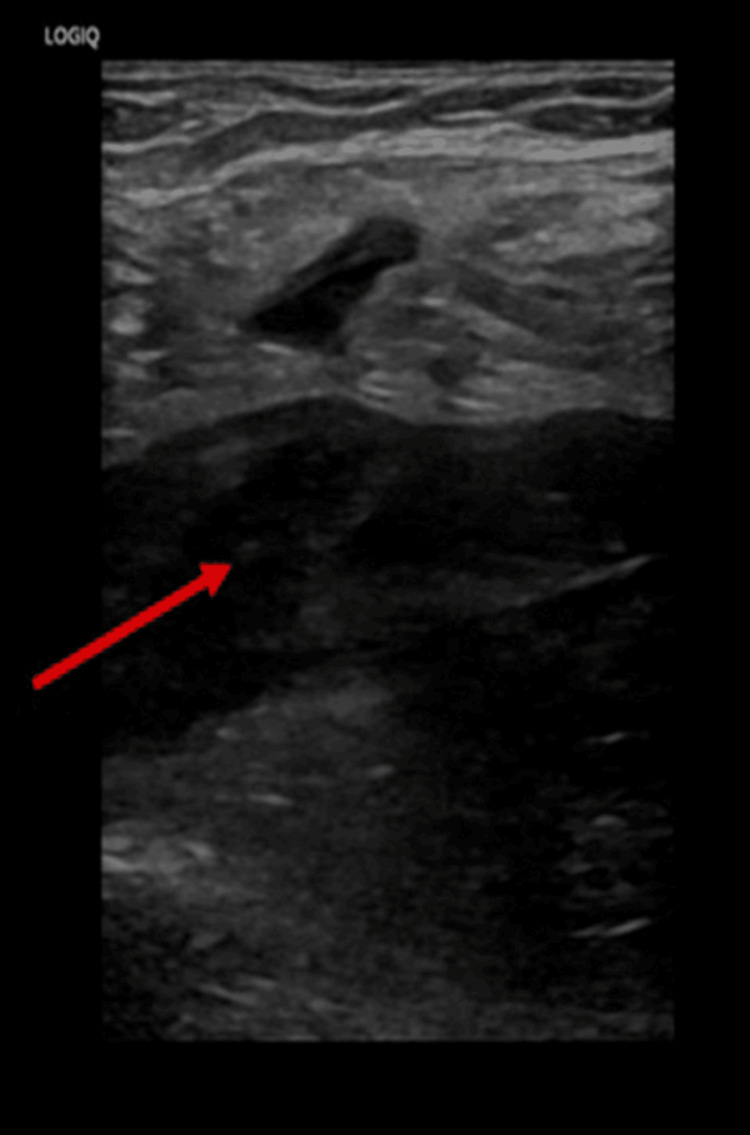
Doppler ultrasound of the left CFV showing a noncompressible vein with intraluminal echogenic thrombus (arrow), consistent with acute deep vein thrombosis CFV: common femoral vein

Initial Doppler ultrasonography of the lower extremities demonstrated extensive DVT. On the right side, thrombi were identified in the external iliac, common femoral, and femoral veins (Figure 1). On the left, there was complete thrombosis extending from the external iliac vein to the posterior tibial veins (Figure 2). Therapeutic anticoagulation was initiated with enoxaparin at a dose of 1 mg/kg administered subcutaneously every 12 hours.

**Figure 3:**
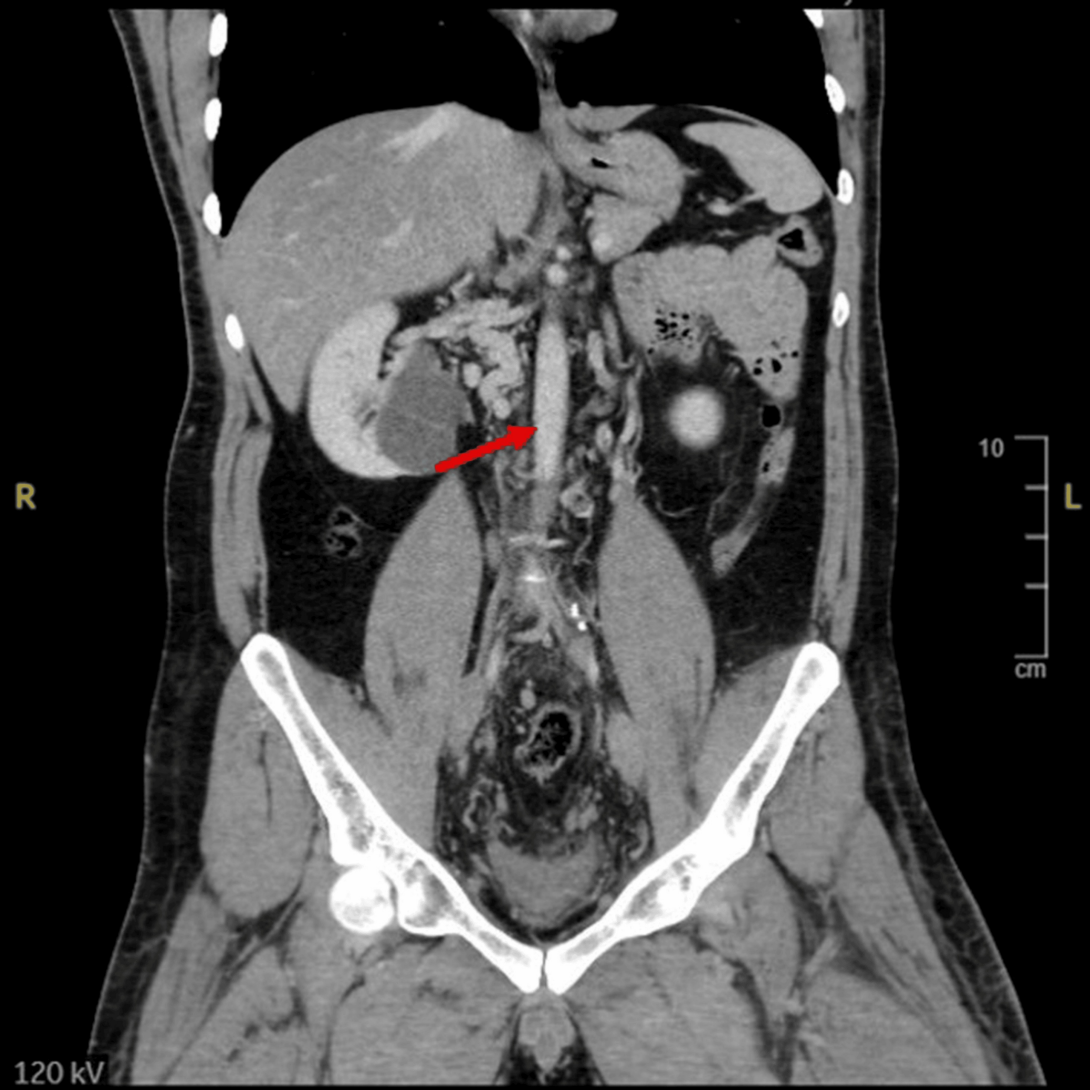
Contrast-enhanced CT of the abdomen demonstrating absence of the inferior vena cava with extensive collateralization CT: computed tomography

**Figure 4:**
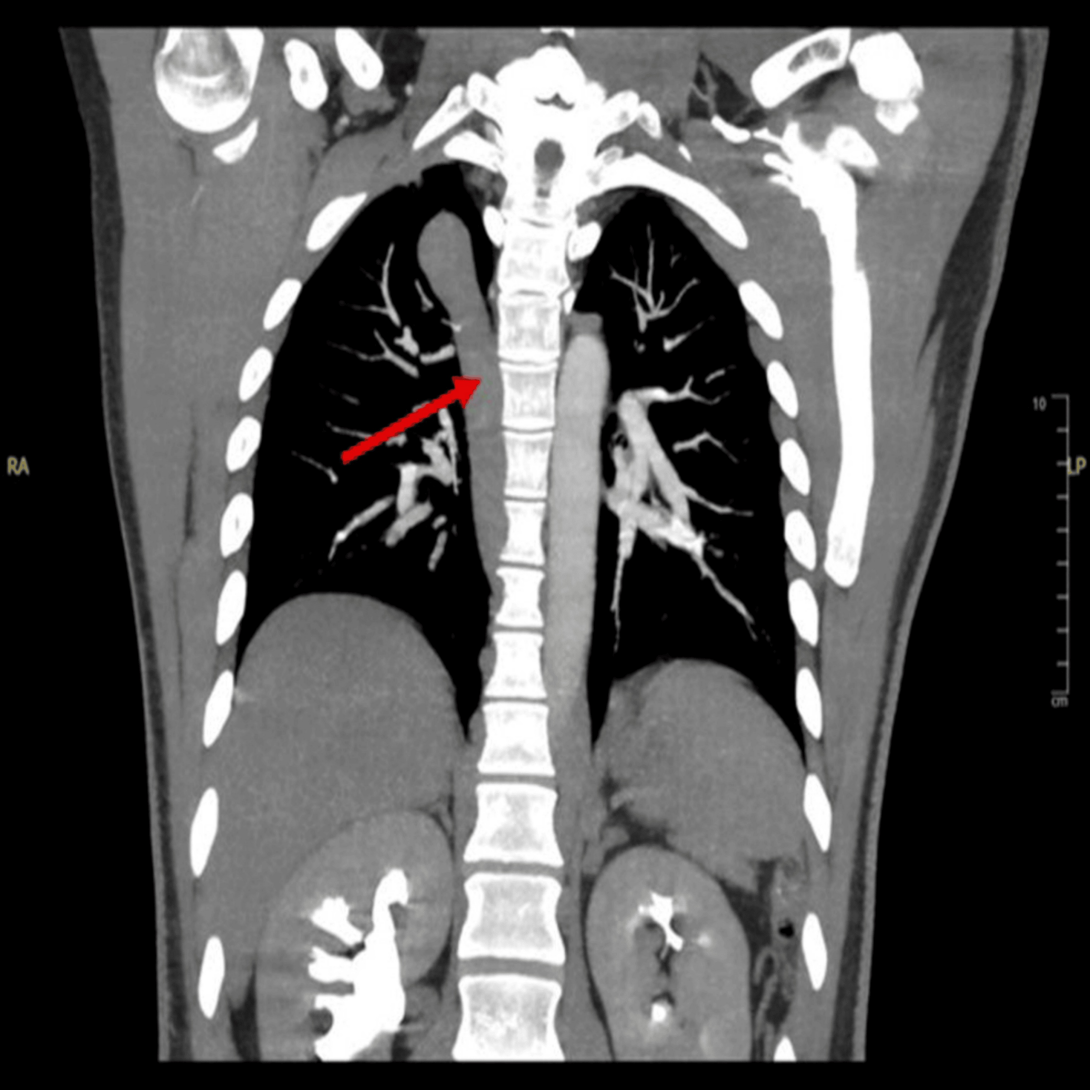
Contrast-enhanced CT of the abdomen demonstrating prominent collateral venous circulation through the azygos/hemiazygos system CT: computed tomography

Given the extent of thrombosis and the patient’s relatively young age, an underlying structural abnormality and inherited and acquired thrombophilia were suspected. Contrast-enhanced CT of the abdomen was performed. The subhepatic segment of the IVC was not visualized, raising suspicion for IVC agenesis (Figure 3). Additionally, thrombosis was identified in the left hemiazygos vein (Figure 4) and both external iliac veins, further supporting the diagnosis of IVC agenesis with associated collateral venous circulation and extensive thrombotic involvement.

Subsequent CT pulmonary angiography confirmed bilateral lower segmental pulmonary emboli. An echocardiogram was performed to assess for right ventricular strain (RVS). No evidence of RVS was observed; however, an incidental bicuspid aortic valve (type 1 Sievers) was identified, with fusion of the right coronary cusp and left coronary cusp (LCC), along with mild aortic stenosis and mild aortic regurgitation.

A multidisciplinary discussion involving interventional radiology and vascular surgery teams concluded that catheter-directed thrombolysis was not indicated, as there was no clinical or radiological evidence of phlegmasia alba dolens or phlegmasia cerulea dolens, and the patient remained hemodynamically stable.

The patient was initially maintained on enoxaparin (1 mg/kg twice daily), later transitioned to fondaparinux 7.5 mg once daily, and subsequently switched to a direct oral anticoagulant for long-term thromboprophylaxis. A summary of the patient’s hematologic and biochemical parameters is presented in Table 1.

**Table 1:** A summary of the patient’s hematologic and biochemical parameters ANA: antinuclear antibody; INR: international normalized ratio; NT-proBNP: N-terminal pro-brain natriuretic peptide; ALT: alanine transaminase; AST: aspartate transaminase

Laboratory test	Result	Reference range
Beta-2 glycoprotein antibodies (IgM, IgG)	<9.00	≤20.00 U/mL
Anticardiolipin antibodies (IgM, IgG)	<9.00	≤20.00 units
Lupus anticoagulant	Negative	-
Antithrombin III	77	80.00%-120.00%
Factor V Leiden mutation	Negative	-
Human leukocyte antigen B51	Positive	-
ANA	Negative	-
Hemoglobin (g/dL)	12.6	13.5-18.0
White cell count (×10^9^/uL)	13.40	3.90-11.00
Platelet count (×10^9^/uL)	238	150-450
Hematocrit (%)	37	37-52
Mean cell volume	90.4	75-95
Neutrophil count (×10^9^/L)	10.20	1.35-7.50
Lymphocyte count (×10^3^/uL)	1.54	1.50-4.30
Prothrombin time (seconds)	16.2	11.9-15.9
INR	1.19	0.87-1.16
NT-proBNP	24.0	≤300.0 pg/mL
Creatinine (umol/L)	73	64.00-104.00
Urea (umol/L)	5.60	3.20-7.40
Potassium (mmol/L)	3.40	3.50-4.50
Na	137.0	136.00-145.00 mmol/L
ALT	15.0	0.00-55.00 U/L
AST	16	5.00-34.00 U/L

## Discussion

This case describes a 35-year-old man presenting with an unusual combination of bilateral DVT, significant lower limb swelling, and severe back pain, ultimately found to have IVC agenesis. Despite the absence of known thrombophilia or traditional risk factors for VTE, the patient developed extensive thrombosis complicated by pulmonary embolism (PE).

Although the underlying anatomical cause was evident, a thrombophilia workup was performed. The results were unremarkable and did not yield clinically significant findings. It is important to note that antithrombin III levels may be falsely reduced during the acute phase of VTE, which should be considered when interpreting such results [8,9]. In this case, thrombophilia testing was conducted primarily for research purposes rather than clinical necessity and did not influence management decisions.

Behçet’s disease was also considered; however, the patient did not meet the diagnostic criteria, a conclusion further supported by rheumatology assessment. Additionally, the patient was referred to cardiology for outpatient follow-up of the incidental echocardiographic finding.

Although the patient tested positive for human leukocyte antigen (HLA)-B51, this marker alone is not diagnostic of Behçet’s disease. In this case, Behçet’s disease was considered unlikely, as the patient denied a history of oral or genital ulcers, skin lesions, or other systemic features. Therefore, the HLA-B51 finding was interpreted as incidental and not contributory to the patient’s thrombotic presentation.

IVC agenesis is a rare congenital anomaly that is often asymptomatic and incidentally detected. However, it can significantly alter venous return by promoting the development of collateral circulation, which predisposes patients to venous stasis and subsequent thromboembolic events [10]. The reported prevalence ranges from 0.0005% to 1% in the general population. IVC anomalies are identified in approximately 0.3%-0.5% of otherwise healthy individuals and up to 0.6%-2% in patients with associated cardiovascular abnormalities [3,11].

IVC anomalies are frequently associated with other congenital conditions, including transposed abdominal viscera, polysplenia or asplenia, renal hypoplasia or agenesis, and pulmonary dysgenesis [12]. Cardiac anomalies such as dextrocardia, atrial septal defects, atrioventricular canal defects, and pulmonary artery stenosis have also been reported in association with infrahepatic interruption of the IVC [12]. In this case, echocardiography revealed an incidental bicuspid aortic valve (type 1 Sievers), characterized by fusion of the right and LCCs, along with mild aortic stenosis and mild aortic regurgitation.

A retrospective study from Taiwan involving 34 patients with IVC interruption demonstrated that only a small proportion had normal cardiac anatomy, while the majority exhibited either simple or complex congenital heart defects [11]. These findings support the concept that IVC anomalies may reflect broader disruptions in embryological development, in which venous and cardiac structures develop concurrently [13].

The presentation in this case is notable for the patient’s young age, absence of clear provoking factors, and rapid development of extensive bilateral DVT with PE. Collectively, these features highlight the likely contribution of IVC agenesis as a predisposing factor for thrombosis [3]. Recognizing this association is clinically important, as it may influence long-term management strategies, including consideration of extended or lifelong anticoagulation, optimization of thromboprophylaxis practices, and, where applicable, alignment with institutional policies and clinical guidelines [14].

Finally, this case underscores the importance of maintaining a high index of suspicion for VTE in patients presenting with atypical symptoms such as lower back pain, particularly when accompanied by lower limb swelling. Increased awareness of congenital vascular anomalies such as IVC agenesis may facilitate earlier diagnosis and more appropriate management.

## Conclusions

IVC agenesis is a rare congenital vascular anomaly that can predispose young patients, even in the absence of classical risk factors, to extensive venous thromboembolism. This case highlights the importance of considering underlying congenital venous abnormalities in patients presenting with unexplained bilateral DVT, particularly when symptoms are atypical, such as back or flank pain. Early recognition of this condition is essential, as it facilitates appropriate long-term anticoagulation planning and may reduce the risk of recurrent thromboembolic events. A multidisciplinary approach, supported by comprehensive imaging, remains key to optimizing patient outcomes.
